# Toxicity and bacterial anti-motility activities of the hydroethanolic extract of *Acacia senegal* (L.) Willd (Fabaceae) leaves

**DOI:** 10.1186/s12906-021-03348-5

**Published:** 2021-06-29

**Authors:** René Dofini Magnini, Mathieu Nitiéma, Geoffroy G. Ouédraogo, Sylvain Ilboudo, Alimata Bancé, Hassanata Millogo-Koné, Carole Di Giorgio, Jean-Marie Pagès, Adama Hilou, Anne Davin-Regli

**Affiliations:** 1 UMR_MD1, U-1261, INSERM, SSA, IRBA, MCT, Faculté de Pharmacie, Université Aix-Marseille, 13385 Marseille, France; 2 Département de Médecine et Pharmacopée Traditionnelle/ Pharmacie (MEPHATRA-PH), Institut de Recherche en Sciences de la Santé (IRSS/CNRST), Ouagadougou 03, 03 BP 7047 Burkina Faso; 3 Laboratoire de Biochimie et de Chimie Appliquée (LABIOCA), Université Pr Joseph Ki-Zerbo, Ouagadougou 03, 03 BP 848 Burkina Faso; 4 Unité Mixte de Recherche Internationale - Environnement, Santé et Sociétés (UMI 3189, ESS) CNRST (Ouagadougou, Burkina Faso) / CNRS (Marseille, France)/ UCAD-UGB (Dakar, Sénégal)/ USTTB, Bamako, Mali; 5 Institut Méditerranéen de Biodiversité et d’Ecologie marine et continentale (IMBE), Aix-Marseille Université, UMR CNRS IRD Avignon Université, Campus Timone – Faculté de Pharmacie, 27, boulevard Jean-Moulin, F-13385 Marseille cedex 05, France

**Keywords:** *Acacia senegal*, Oral toxicity, Bacterial motility, Cytotoxicity

## Abstract

**Background:**

*Acacia senegal* is a plant traditionally used for its various properties, including the treatment of infectious diseases. Recently, our team has demonstrated the ability of the hydroethanolic extract of the leaves to increase the activity of phenicol antibiotics against multi-resistant bacteria. The aim of this work is to determine the toxicological effects of the extract and its capacity to inhibit the bacterial mobility of Gram-negative bacteria, in order to evaluate the level of safety use of this plant.

**Methods:**

The cytotoxicity test was performed using the neutral red absorption method. Acute and sub-acute oral toxicity were conducted on NMRI mice and Wistar rats. The behaviour and adverse effects were recorded during the 14 days of the acute study. For the subacute test, biochemical parameters, food and water consumption, and morphological parameters were determined. The anti-motility activities were evaluated on *Pseudomonas aeruginosa* PA01 and *Escherichia coli* AG100, using specific concentrations of Agar as required by the method.

**Results:**

HEASG induced inhibition of keratinocytes cell growth with an IC_50_ of 1302 ± 60 μg/mL. For the acute toxicity study in mice, the single dose of extract of 2000 mg/kg body weight caused no deaths and no behavioural changes were observed; therefore, the median lethal dose (LD_50_) of HEASG was calculated to 5000 mg/kg body weight. In Wistar rats, no mortality was observed at 250, 500 and 1000 mg/kg/day during the 28-day subacute oral toxicity study. The weights of both females and males increased globally over time, regardless of the batch. No statistically significant differences were registered for organ weights and biochemical parameters, except for chloride for biochemical parameters. Water and food consumption did not change significantly. Furthermore, no macroscopic changes in organ appearance were observed. Regarding anti-motility activity, the extract has reduced the swarming motility of PA01 and AG100 significantly at the concentration of 32 μg/mL (*P* < 0.001). The extract has reduced the swimming motility (*P* < 0.01) of PA01 but not AG100.

**Conclusions:**

The results suggest that hydroethanolic extract of *A. senegal* leaves has significant activity against bacterial motility and relatively low toxicity.

**Supplementary Information:**

The online version contains supplementary material available at 10.1186/s12906-021-03348-5.

## Background

Plants have always been very widely used by humans, as a food source and also for their curative/preventive effects against several diseases [1, 2]. Today, medicinal plants present undeniable medicinal interest [3] because of the many natural therapeutic products they produce that can be use in addition face to the continuous increase of resistance towards synthetic molecules such as antibiotics [4, 5]. Moreover, the toxicity associated to the repeated use of synthetic drugs contributes to a greater demand for phytotherapy and natural extracts. *Acacia senegal* is the plant species belonging of the Fabaceae family, well known in African traditional medicine. Traditionally, the bark of trunks, roots and gum are used in Burkina Faso against respiratory infections, influenza and sinusitis, sexually transmitted diseases, diarrhoea, gastric ulcers, haemorrhoids etc. [6, 7]**.** The leaves are used as a feed supplement for cattle [8]. Pharmacological data has shown that ethyl acetate extract from the stem bark of *A. Senegal* causes a significant decrease in blood glucose, Total chlolesterol serum (TC serum), Tissue transglutaminase serum (serum TTG), Low density lipoprotein serum (LDL serum), serum urea and creatinine and increase in serum high density lipoprotein (HDL) on the 16th day after administration in albino rats rendered diabetic to alloxan [9]. Also, the hydroethanolic extract of *A. senegal* pods significantly reduced acute hepatotoxicity induced by carbon tetrachloride (CCL4) in Wistar albino rats [10]. Ethanolic extract from the leaves decreased sucrose enzyme activity, promoted control of carbohydrate hydrolysis, and therefore reduced the increase in postprandial blood glucose levels in diabetic rats [11]. A study carried by Seif MM and collaborators [12] evaluated the efficacy of *Acacia senegal* extracts for the improvement of hepatic and cerebral toxicity induced by Di-2- Ethylhexyl phthalate (DEHP). Sprague Dawley rats, in which acute hepatotoxicity and neurotoxicity were induced by DEHP, were treated orally with a 70% ethanolic extract of *A. senegal* pods for 28 days under several conditions. Results showed that the *A. senegal* extract restored antioxidant enzyme activities to normal, reducing the level of LPO in both tissues. In addition, the extract improved the levels of brain amino acids, monoamines and their metabolites. Methanolic extract from the bark of the stem showed 100% mortality against worms adult *Fasciola gigantica* at concentrations of 1000, 500 and 250 ppm after 6, 12 and 24 h respectively [13]. Research conducted by Mudi and Salisu [14] demonstrated that the hexanic fraction of the bark of the stem of *A. senegal* is active against respiratory pathogenic bacteria, notably *Klebsiella pneumonia* and *Streptococcus pneumoniae*. Furthermore, methanolic and ethanolic extracts of *A. senegal* trunk bark showed antibacterial activity against *K. pneumoniae, Proteus vulgaris*, *Salmonella Typhi*, *Shigella dysenteriae* and *E. coli* and the toxicity studies of the ethanol extracts revealed that they exhibited no significant toxicity (LC_50_ of 100 μg/ml) against *Artemia salina* [15]. Recent studies have shown that *A. senegal* hydroethanolic extract synergizes antibacterial activities of phenicol antibiotics when used in combination against resistant Gram negative bacteria. A permeabilizing effect of the extract on the outer membrane of these multi-drug resistant bacteria was also reported [16]. Also, given the high demand for its gum arabic, and the synergistic activity with the phenicol family proven on multi-resistant bacterial strains, the safety of use and the innocuousness remain little documented. With the importance of *A. senegal* in the field of phytotherapy and the place it occupies in projects (Great Green Wall) involving several Sahelian countries, we perform this work in order to evaluate the possible cytotoxic effects and risks of the acute and subacute toxicity of the hydroethanolic extract of *A. senegal* leaves, and also the capability of this extract to inhibit bacterial motility are determined for evaluating the safety of use of *Acacia senegal* species.

## Methods

### Plant material

Leaves of *Acacia senegal* (L) willd. Were collected in June and August in the area of Saaba to Gonsé, located twenty kilometres from Ouagadougou (Burkina Faso), after validation of the thesis protocole research at University of Joseph Ki-Zerbo of Ouagadougou. The plant identification was made at Université Joseph Ki-Zerbo of Ouagadougou at the Laboratory of Plant Biology and Ecology by Pr. Amadé Ouedraogo, where the voucher specimen is deposited under the reference number 6896/17257. The leaves were dried at ambient temperature under ventilation in the shade for 2 weeks. The dried leaves were ground and stored for extraction.

### Preparation of the extract

One hundred grams of *Acacia senegal* leaves powder was macerated with petroleum ether (500 ml) at room temperature for 24 h as a first step. Afterwards, the mixture was filtered through the Whatman No.1 filter; the marc was dried and macerated again in 70% ethanol (V/V) overnight. The supernatant was collected, concentrated and frozen before being lyophilized to obtain the powder of *A. senegal* extract (HEASG).

### In vitro cytotoxicity evaluation

HaCaT (human Squamous Cell Carcinoma from Tongue) cells (ATCC® CRL-1624™) were obtained from ATCC® culture collection, cultured in Dulbecco’s modified Eagle’s medium containing 10% fetal bovine serum and penicillin (100 IU/ml)/streptomycin (100 μg/ml) (Invitrogen, Carlsbad, CA, USA), under a humidified atmosphere of 5% CO_2_ at 37 °C.

Human keratinocytes (HaCaT) were seeded into 96-well tissue culture plates (0.2 mL per well), at 1.10^5^ cells/mL [17], and incubated at 37 °C (5% CΟ_2_) for 24 h until semi-confluent. The culture medium was decanted and replaced by 200 μL of complete medium containing the appropriate concentrations of the hydroethanolic extract of *A. senegal* leaves (8 different concentrations), then cells were incubated at 37 °C (5% CO_2_) during 24 h. After incubation, the culture medium was removed. The cells are washed and placed in Neutral Red medium (50 μg/mL of Neutral Red in the complete medium) and incubated for 3 h at 37 °C (5% CO_2_). Then the medium is removed, and the cells are washed three times with 0.2 mL HBSS to remove excess dye. The neutral red medium was removed, and the staining solution (50% ethanol, 1% acetic acid, 49% distilled water; 50 μL per well) was added to the wells. The plates were shaken for 15–20 min at room temperature in the dark [18]. Αll the test samples and controls were run in triplicates, in independent experiments. A fluorescence-luminescence reader Infinite M200 Pro (TECAN) measured the degree of membrane damage (i.e. the increase of released Neutral Red). The, of each well was read at 540 nm. The results obtained for wells treated with HEASG were compared to those of untreated control wells (HBSS, 100% viability) and converted to percentage values [19]. The concentration of HEASG, causing 50% release of the preloaded Neutral Red as compared to the control culture, was calculated using software Graph Pad Prism 5.0. The mean absorbance value of the blank wells (containing only the desorbed Neutral Red solution) was subtracted from the mean OD value of three treated wells (dilutions of extract, positive control or HBSS). The cell viability percentages were estimated as follows:

$$ \mathrm{Viability}\ \left(\%\right)=\frac{\mathrm{Mean}\ \mathrm{OD}\ \mathrm{of}\ \mathrm{test}\ \mathrm{wells}-\mathrm{mean}\ \mathrm{OD}\ \mathrm{of}\ \mathrm{blanks}}{\mathrm{Mean}\ \mathrm{OD}\ \mathrm{of}\ \mathrm{negative}\ \mathrm{control}-\mathrm{mean}\ \mathrm{OD}\ \mathrm{of}\ \mathrm{blanks}} $$

### Experimental animals

Healthy Female NMRI mice (5–8 week-old and weighing 22–29 g) and Wistar rats (7–10 week old and weighing 134–204 g) were used in the study, respectively. They were obtained from the pet shop of Institut de Recherche en Sciences de la Santé (IRSS), Ouagadougou, Burkina Faso. The selected animals were kept in their plastic cages for 6 days for acclimatization before testing began under normal laboratory conditions (12 h light/dark cycle and 25 ± 2 °C). Water and laboratory pellets, enriched with 29% protein, were freely accessible to the animals. The experimental protocol was carried out following international standard protocols [Guidelines set by the European Union on the protection of animals (CEC Council 86/609)] and adopted by IRSS, Burkina Faso [20, 21]. All sections of this report adhere to the ARRIVE guidelines for reporting animal research **(**Additional file 1**).**

### Acute toxicity test

The acute toxicity test was carried out following to OECD test guideline 423 for acute oral toxicity [22]. After a 4 h fastening period, the mice were weighed, and the dose of HEASG was calculated from the body weight. HEASG was administered orally by gavage in a single dose to the mice according to a sequential procedure. In performing the test, 2000 mg/kg b.w. of HEASG was used as a starting dose. Two hours after treatment, all animals were observed, and feeding was re-established. They are then observed at least once daily for 14 days for mortality and signs of toxicity such as changes in skin and fur, eyes, mucus membranes, salivation, convulsion, diarrhoea, lethargy, sleep, and coma [20, 21].

### Sub-acute toxicity study

This test was carried in accordance with the OECD test guideline 407 [23]. Briefly, forty rats were randomly selected including 20 females and 20 males. Females involved were nulliparous and non-pregnant. The rats were divided into four groups of 10 animals each (5 males and 5 females); males and females were placed separately in polypropylene cages. Group 1 served as a control and received the control (distilled water), while the rats in Group 2, 3 and 4 were respectively received daily doses of 250, 500 and 1000 mg/kg b.w of the HEASG for 28 days at the same hour. The animals were observed during the first 1 and 4 h of dosing to examine all adverse toxic markers, behavioural variation and at least twice a day for mortality and morbidity. Body weight and food consumption were reported once weekly. Water consumption was monitored daily for each cage (5 rats per cage) up to 4 weeks. On the 29th day, after overnight fastening, we have proceeded to animals sacrifice. The rats generally anesthetized by intraperitoneal injection of 150 mg/kg of ketamine. After 10 to 30 min of ketamine administration, the animals were completely unconscious. They were then placed in the supine position and the abdominal cavity of each rat was opened. Blood samples were collected by cardiac puncture using a 5 mL syringe, followed in animals death within 5 min. Then vital organs of animals were isolated [20].

### Determination of the relative weight of organs

At the end of HESAG treatment, after overnight fastening, all animals were sacrificed, and vital organs such as heart, kidneys, liver, lung, gonads (testis or ovaries) and spleen were isolated and observed macroscopically for any lesions. After that, all organs were dried using hygienic paper and then weighed on a precision balance (Sartorius; precision 0.1 mg). The relative organ weight ratio (ROW) of each rat was determined as follows [24]:

$$ \mathrm{ROW}\ \left(\%\right)=100\ \mathrm{X}\frac{\mathrm{Absolute}\ \mathrm{organ}\ \mathrm{weight}\ \left(\mathrm{g}\right)}{\mathrm{Body}\ \mathrm{weight}\ \mathrm{of}\ \mathrm{rats}\ \mathrm{on}\ \mathrm{sacrifice}\ \mathrm{day}\ \left(\mathrm{g}\right)} $$

### Biochemical parameters

The blood samples collected in dry vacutainers were centrifuged at 3000 rpm for 10 min using a centrifuge (ROTOFIX 32A, Mettich Zenfrifugen, Germany); the serum obtained was used for biochemical assays. Blood chemicals tests were carried out using an automatic biochemistry analyzer (Mindray BS-300, China). Biochemical parameters, including total proteins, aspartate aminotransferase, alanine aminotransferase, creatinine, cholesterol, fasting blood glucose, chloride, phosphorus, and magnesium, were determined.

### Anti-motility activities

#### Bacterial strains

The phenotypic and/or genotypic characteristics and minimum inhibitory concentrations are reported in Table 1.

**Table 1:** Bacterial strains used in this study

Strains	Phenotype	Reference	MIC (mg/L) HEASG
PA01	*P. aeruginosa*, Wild type	[25]	> 512
AG100	Parental *E. coli* K-12 Porin+; basal efflux	[26]	256

*MIC* Minimal Inhibitory Concentration, *HEASG* hydroethanolic *Acacia senegal* leaves

#### Swimming and swarming motility assays

The bacterial strains used in our study are obtained from the laboratory collection of research unit UMR-MD1/MCT, INSERM U1261 of the Aix Marseille University, France. They are stocked in cryotubes containing a 15% glycerol solution and stored in a freezer - 80.

For swimming motility, 0.3% Agar containing 10 g tryptone, 5 g yeast extract and 0.5% NaCl was used [27]. For swarming motility, LB medium supplemented with 5 g de glucose and 5 g of Agar were used [28]. The plant extracts were added to the motility Agar, and DMSO (0.1%) was added as a control. PA01 and AG 100 were grown to an OD_600_ of 1.0, and ~ 0.2 μL of culture was placed on the motility plates using a sterile pipette tip. The diameters of the swimming and swarming halos were measured after 24 h. Each experiment was repeated using three independent cultures.

### Statistical analysis

Statistical analysis was done using ANOVA. Results are shown as mean ± SD of three determinations. The statistical value of the difference between the treatment and control groups was interpreted by one-way analysis of variance (ANOVA) using Graph Pad Prism 5 (Graph Pad Software, San Diego, CA, USA) followed by Dunnett’s multiple comparison tests. Significant differences in treatment were accepted at *P* < 0.05.

## Results

### Cytotoxicity evaluation

**Fig. 1:**
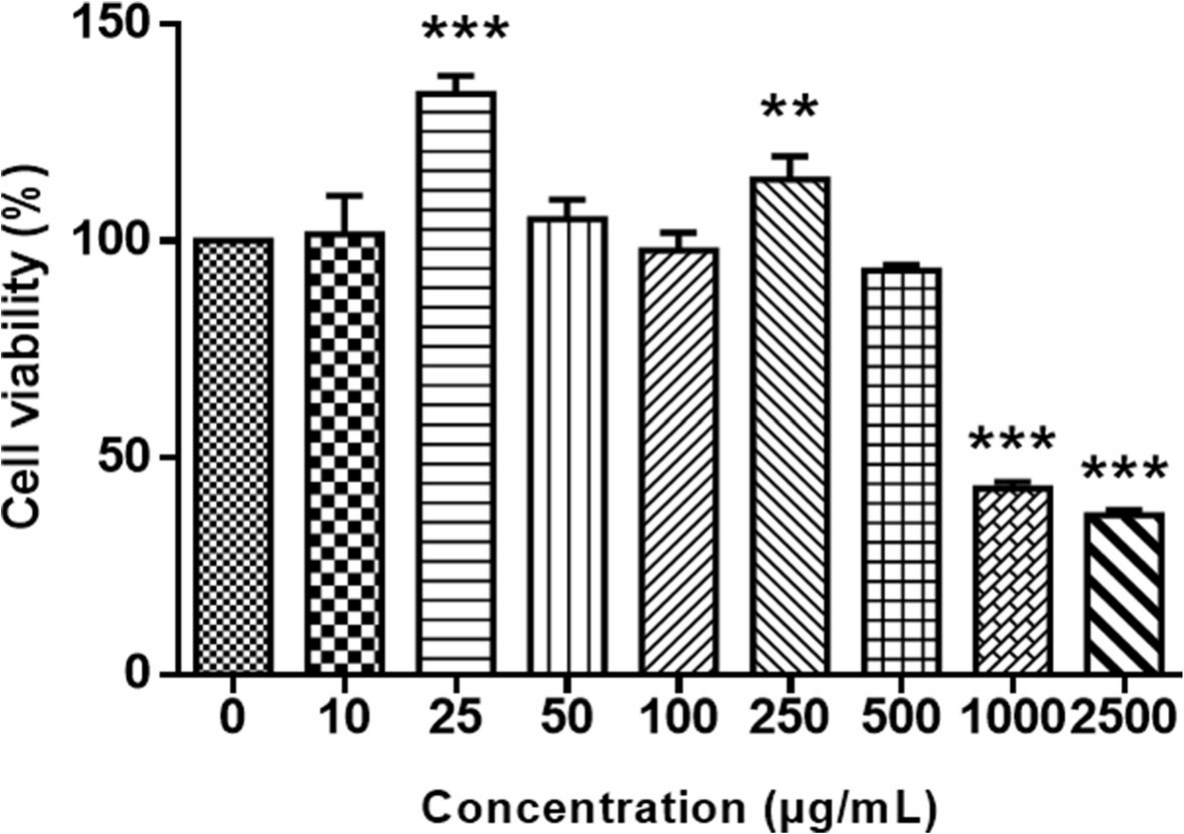
Effect of different concentration of HEASG on the viability of human keratinocyte cells HaCat. The values are expressed as mean ± SD of three independent experiments. *** indicated *p* < 0.001, ** indicated *p* < 0.01 versus the untreated control; *n* = 3

The percentage viability of HaCaT cells in the presence of HEASG was determined from OD measurements **(**Fig. 1). The different concentrations of HEASG increased cell viability up to the dose of 250 μg/mL. The cytotoxic effects of HEASG were clearly observed at 1000 and 2500 μg/mL doses, compared to the control with a significant difference (*P* < 0.001). This reflects a strong decrease in the relative amount of live HaCaT cells with an IC_50_ (50% inhibitory concentration) equal to 1302 ± 60 μg/mL.

### Acute toxicity study of *Acacia senegal* leaves hydroethanolic extract in mice

During the 14 days of observation post-treatment, no deaths were observed in the groups of experimental animals. Per os administration of a single dose of 2000 mg/kg of HEASG did not induce any significant changes in the mice. According to the acute toxicity class method, HEASG tested can be classified to the 5th toxicity class with an LD_50_ value estimated to 5000 mg/kg b.w.

### Sub-acute toxicity study

The results obtained from subacute toxicity study with daily administration at repeated doses (250, 500 and 1000 mg/kg) of HEASG allowed the evaluation of behavioural parameters, water and food consumption, relative weight growth, relative organs weights and biochemical parameters.

### Body weight

**Fig. 2:**
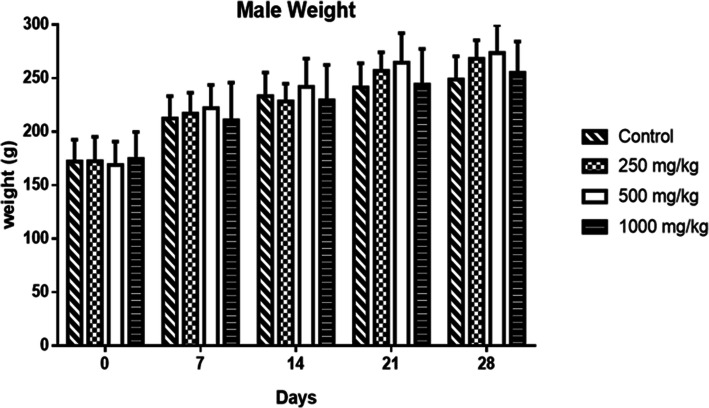
Mean absolute weight of control and treated males rats groups with different doses of HEASG. Mean and Standard deviation are presented (*n* = 5)

**Fig. 3:**
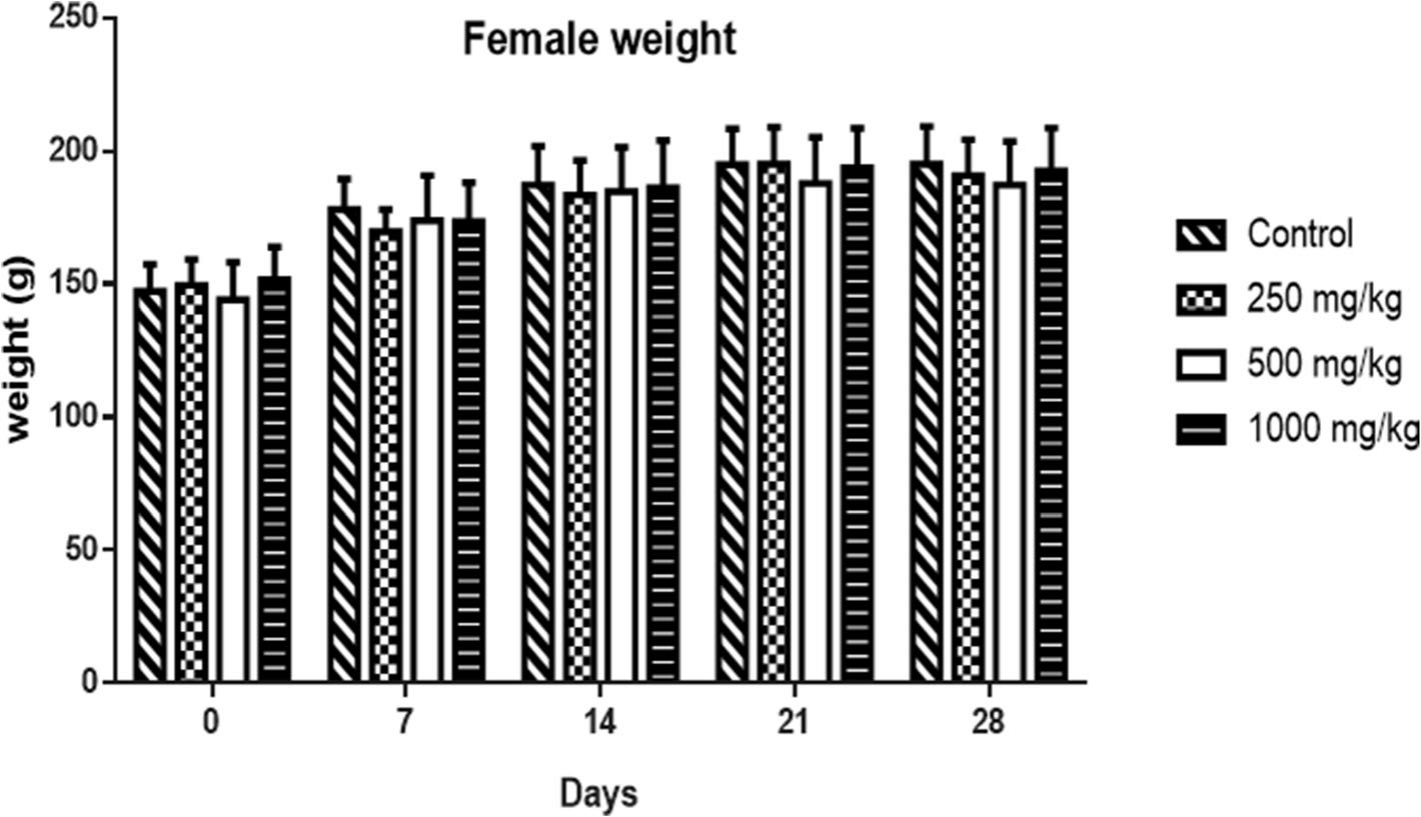
Mean absolute weight of control and treated females rats groups with different doses of HEASG. Mean and Standard deviation are presented (*n* = 5)

At all doses of HEASG tested, no significant behavioural changes were observed in males or females. The weights of males **(**Fig. 2**)** and females **(**Fig. 3**)** increased in all groups over time. Statistical analysis indicated any significant difference between the treated and the control groups.

### Water intake and food consumption

Table 2 summarizes water consumption data for the 28 days of observation. No significant variation was observed between control and treated animals groups. Table 3 provides information on food consumption at each dose level compared to the control group. Treated animals at the different doses compared to the control group showed no significant variation in either males or females.

**Table 2:** Mean daily water intake (mL/day/rat) during 28 days of treatment with HEASG

Dose of HEASG	SEX	Week 1	Week 2	Week 3	Week 4
Control	F	35 ± 7	35 ± 7	30 ± 5	31 ± 3
	M	48 ± 14	42 ± 8	37 ± 2	39 ± 6
250 mg/kg	F	30 ± 5	31 ± 2	27 ± 5	32 ± 3
	M	47 ± 11	44 ± 8	36 ± 5	40 ± 6
500 mg/kg	F	34 ± 10	32 ± 2	28 ± 2	28 ± 1
	M	52 ± 13	44 ± 10	39 ± 6	42 ± 4
1000 mg/kg	F	31 ± 4	31 ± 3	27 ± 6	33 ± 5
	M	43 ± 10	41 ± 9	40 ± 3	43 ± 5

Mean and standard déviation are reported as follows (*n* = 10; 5/sex). *M* Male, *F* Female

**Table 3:** Mean daily food consumption (g/day/rat) during 28 days of treatment with hydroethanolic extract of *A. senegal* leaves

Dose of HEASG	SEX	Week 1	Week 2	Week 3	Week 4
Control	F	20.71	19.71	19.80	17.20
	M	22.88	22.14	21.42	20.28
250 mg/Kg	F	17.45	18.02	17.94	16.68
	M	23.20	24.51	21.42	23.20
500 mg/Kg	F	17.54	16.80	16.25	16.78
	M	24.91	26.80	24.45	21.85
1000 mg/Kg	F	17.02	17.34	16.00	15.57
	M	18.14	21.02	26.51	21.34

Mean and standard déviation are reported as follows (*n* = 10; 5/sex). *M* Male, *F* Female

### Effect of HEASG on organs weights in rats

Table 4 showed the effect of the toxicity of HEASG on mean vital relative organ weights in rats. Compared to the mean weight of controls, HEASG did not cause any significant change in mean organ weights in treated rats (p<0.05).

**Table 4:** Mean relative organ weights (%) of rats after 28 days of treatment with HEASG

Organs	Sex	Control	250 mg/Kg	500 mg/Kg	1000 mg/Kg
Lung	F	0.55 ± 0.04	0.56 ± 0.08	0.52 ± 0.04	0.54 ± 0.12
	M	0.53 ± 0.02	0.46 ± 0.03	0.45 ± 0.03	0.49 ± 0.07
Kidney	F	0.65 ± 0.03	0.67 ± 0.08	0.64 ± 0.05	0.63 ± 0.06
	M	0.64 ± 0.05	0.65 ± 0.05	0.64 ± 0.05	0.64 ± 0.07
Spleen	F	0.23 ± 0.02	0.21 ± 0.02	0.23 ± 0.03	0.23 ± 0.04
	M	0.19 ± 0.03	0.20 ± 0.03	0.17 ± 0.02	0.20 ± 0.10
Liver	F	3.20 ± 0.60	3.00 ± 0.30	2.90 ± 0.30	3.00 ± 0.30
	M	2.50 ± 0.10	2.90 ± 0.20	2.80 ± 0.20	2.90 ± 0.30
Heart	F	0.34 ± 0.03	0.33 ± 0.03	0.35 ± 0.02	0.31 ± 0.04
	M	0.35 ± 0.03	0.33 ± 0.02	0.33 ± 0.02	0.31 ± 0.04
Gonads	F	0.06 ± 0.01	0.05 ± 0.02	0.07 ± 0.02	0.06 ± 0.02
	M	1.20 ± 0.10	1.17 ± 0.04	1.23 ± 0.12	1.10 ± 0.10

Mean and standard deviation are represented (*n* = 10; 5/sex). *M* Male, *F* Female. *p* < 0.05

### Effect of HEASG on biochemical parameters of rats

Daily oral administration repeated at doses of 250, 500 and 1000 mg/kg of HEASG allowed the evaluation of biochemical parameters. Examination of serum electrolytes did not show statistically significant variation between the treated female or male groups and their respective control groups **(**Table 5). These results indicated that orally administration of HEASG at doses of 250, 500 and 1000 mg/kg b.w on rats for 28 consecutive days did not cause statistically significant changes in blood serum biochemical parameters such as sodium, potassium, calcium, phosphorus, aspartate aminotransferase (AST), alanine aminotransferase (ALT), total proteins, fasting blood glucose, creatinine and total cholesterol levels when compared to control groups. However, a statistically significant difference in chloride level was observed in the group treated at a dose of 250 and 500 mg/kg b.w. when compared to control groups (*P* < 0.01) in both sexes.

**Table 5:** Biochemical parameters of rats after 28 days of treatment with HEASG

Biochemical parameters	Sex	Control	250 mg/Kg	500 mg/Kg	1000 mg/Kg
Sodium (mmol/L)	F	139.4 ± 4.5	143.2 ± 5.9	139.0 ± 6.8	150.4 ± 2.5
	M	139.2 ± 3.1	141.0 ± 3.3	146.23 ± 2.4	146.7 ± 1.3
Chloride (mmol/L)	F	121.0 ± 3.0	104.7 ± 1.5*	105.3 ± 4.0*	116.3 ± 6.4
	M	122.0 ± 2.1	100.8 ± 3.3*	104.8 ± 1.0*	122.0 ± 3.0
Potassium (mmol/L)	F	5.4 ± 1.0	5.6 ± 1.2	5.0 ± 0.4	5.0 ± 0.6
	M	5.8 ± 0.8	5.9 ± 0.4	5.0 ± 0.5	5.8 ± 0.6
Calcium (mmol/L)	F	3.5 ± 0.2	3.1 ± 0.1	3.1 ± 0.4	3.3 ± 0.2
	M	3.4 ± 0.2	3.0 ± 0.1	3.1 ± 0.3	3.2 ± 0.1
Phosphorus (mmol/L)	F	3.9 ± 0.6	3.8 ± 0.3	3.5 ± 0.3	3.9 ± 0.6
	M	4.5 ± 0.4	4.2 ± 0.3	3.7 ± 0.4	4.2 ± 0.3
ASAT (U/L)	F	133.7 ± 42	123.0 ± 30	129.8 ± 58	130.0 ± 34
	M	129.0 ± 17	112.0 ± 28	129.0 ± 31	120.6 ± 27
ALAT (U/L)	F	36.5 ± 6.8	35.5 ± 3.1	34.3 ± 9.8	39.0 ± 9.4
	M	43.6 ± 6.2	40.8 ± 6.5	38.0 ± 3.0	46.2 ± 6.0
Total protein (g/L)	F	61.8 ± 4.2	63.8 ± 4.1	62.5 ± 3.3	64.6 ± 3.1
	M	61.5 ± 2.9	56.9 ± 1.2	61.0 ± 2.0	65.5 ± 3.1
Fasting blood glucose (mmol/L)	F	6.2 ± 0.8	4.6 ± 0.6	5.4 ± 0.1	5.4 ± 1.1
	M	4.5 ± 0.7	4.4 ± 0.9	4.3 ± 1.2	4.4 ± 0.6
Creatinine (μmol/L)	F	67.5 ± 0.5	64.7 ± 2.9	62.0 ± 2.7	66.8 ± 1.2
	M	60.1 ± 5.2	60.6 ± 6.3	56.8 ± 3.9	61.7 ± 4.9
Total cholesterol (mmol/L)	F	1.30 ± 0.30	1.20 ± 0.40	1.10 ± 0.05	1.30 ± 0.30
	M	1.70 ± 0.20	1.30 ± 0.03	1.42 ± 0.05	1.50 ± 0.30

Mean and standard deviation are represented (*n* = 10; 5/sex). *M* Male, *F* Female, * indicate statistically significant difference in values (*p* < 0.05) compared to control. Aspartate aminotransferase (AST); alanine aminotransferase (ALT)

### Motility assays

**Fig. 4:**
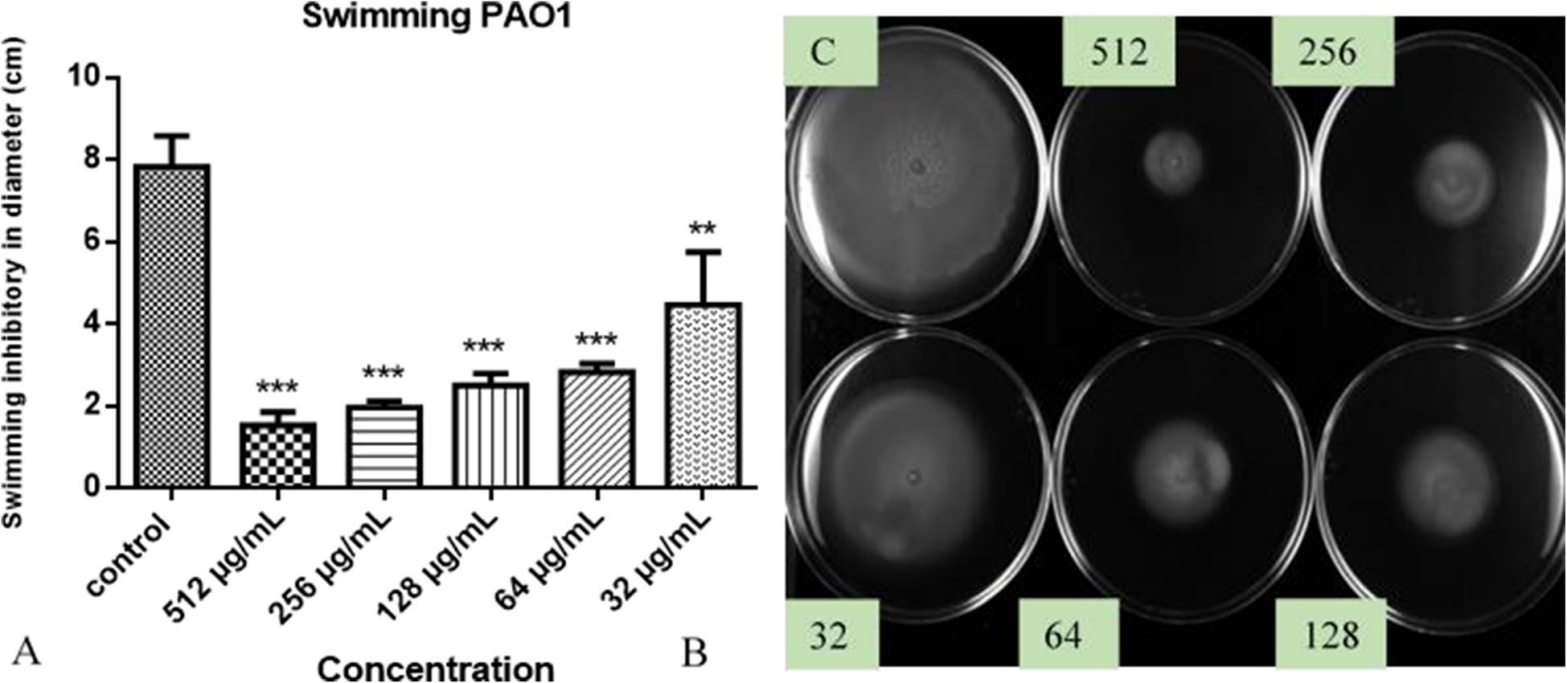
**A** Effect of HEASG on PA01 motility. **B** HEASG inhibited swimming motility. Each experiment was carried using three independent cultures, and one representative data set is shown. ** indicated *p* < 0.01 and *** indicated *p* < 0.01 versus the untreated control

The effects of the hydroethanolic extract at sub-MIC levels (32 to 512 μg/mL) on the swimming, and swarming motilities of PA01 and AG100 were investigated. The extract has reduced swimming and swarming motility in comparison to the control (no treated). More interestingly, the different concentrations of HEASG had variable impacts on swimming and swarming motilities. Swimming motility of PA01 was inhibited with values corresponding to approximately 43 and 64% of control at concentrations of 32 and 64 μg/mL, respectively (Fig. 4A).

**Fig. 5:**
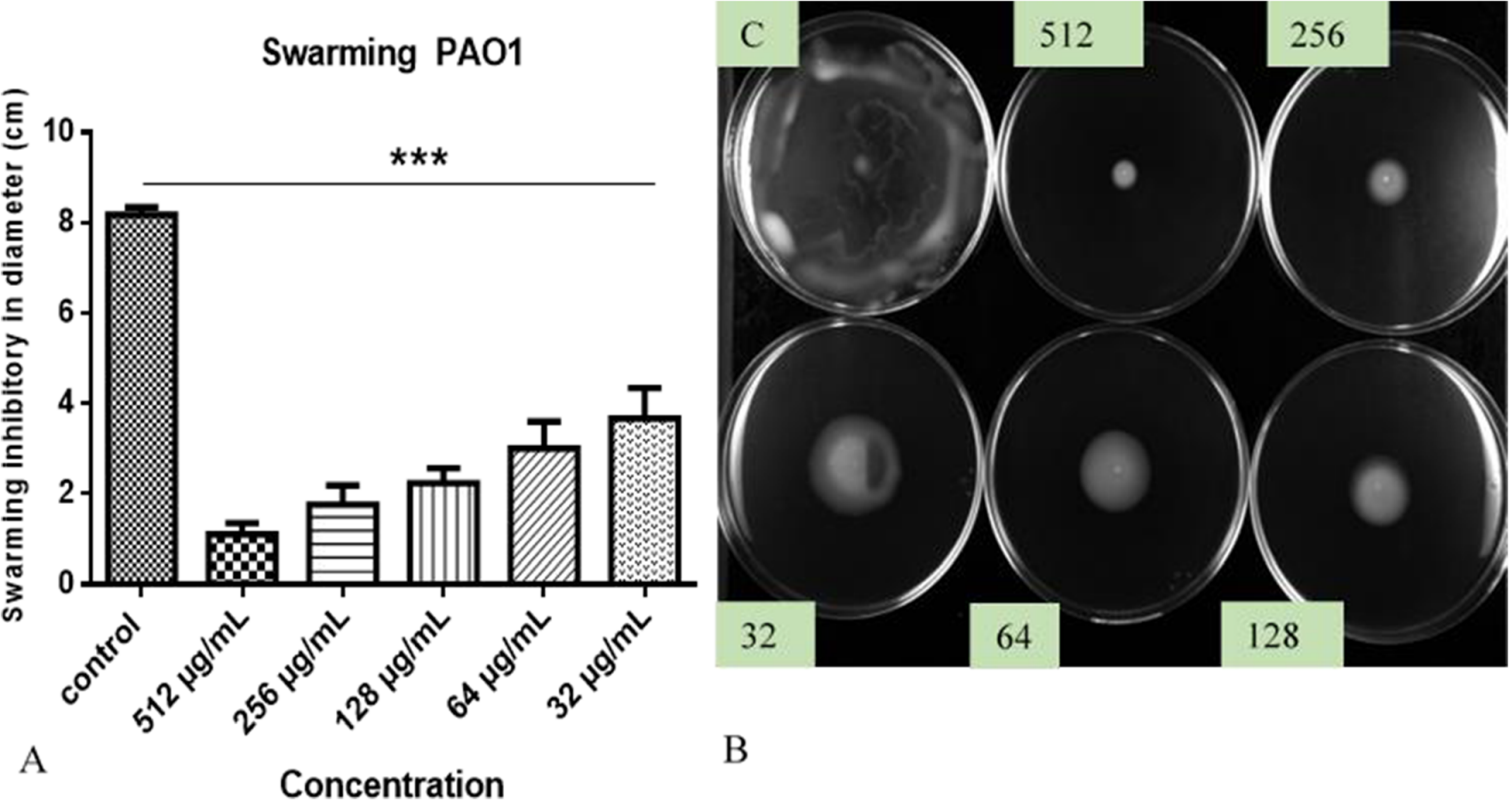
**A** Effect of HEASG on PA01 motility. **B** HEASG inhibited swimming motility. Each experiment was carried using three independent cultures, and the representative values are presented. *** indicated *p* < 0.01 and indicated *p* < 0.001 versus the untreated control

The swarming motility of PA01 also decreased with level of 55 and 63% compared to control at concentrations of 32 and 64 μg/mL, respectively (Fig. 5A).

**Fig. 6:**
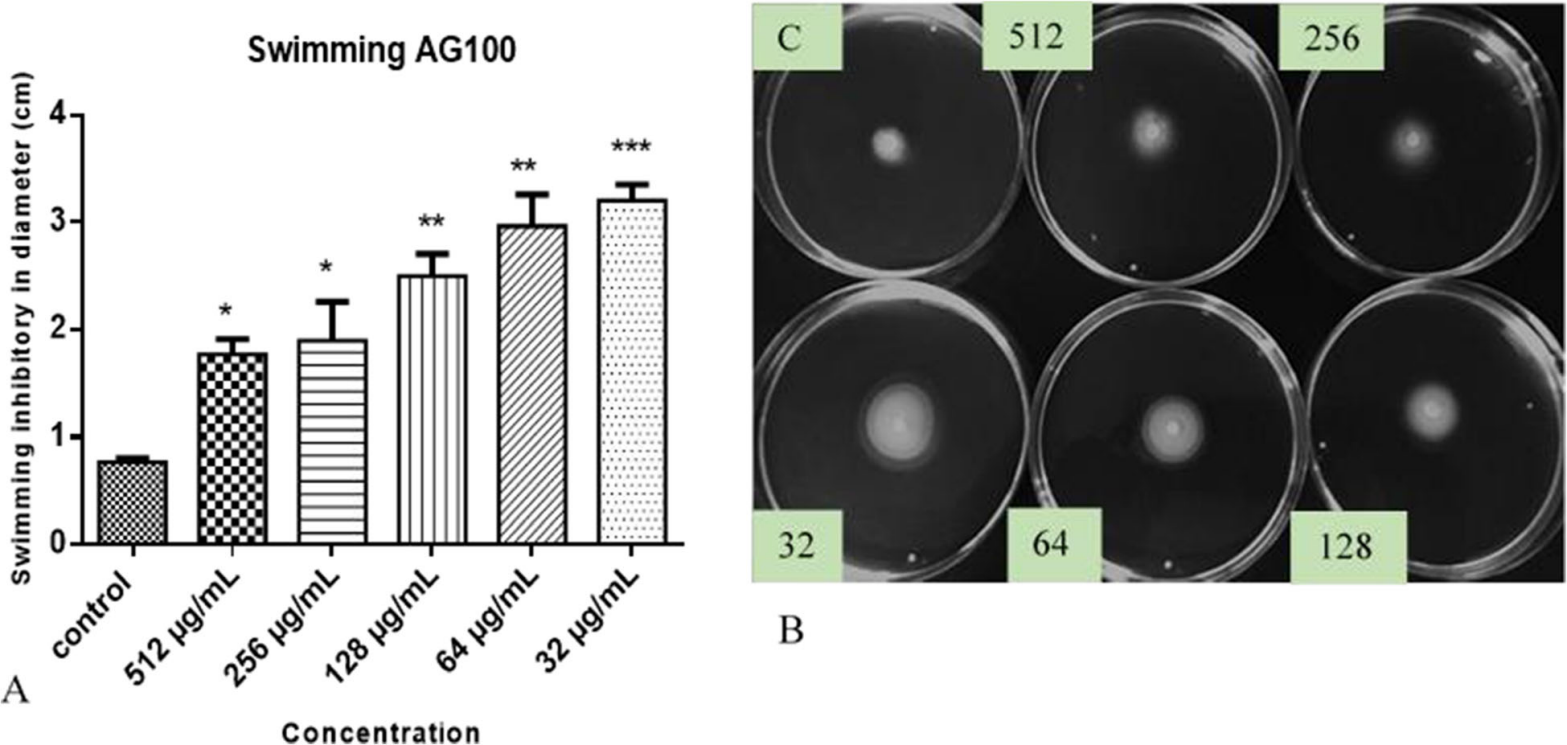
**A** Effect of HEASG on AG100 swimming motility, **B** HEASG inhibited swimming motility, respectively. Each experiment was carried using three independent cultures, and the representative values are presented. The results are expressed as mean ± SD. *n* = 3; *** indicated *p* < 0.001, ** indicated *p* < 0.01 and * indicated *p* < 0.05 versus the untreated control

**Fig. 7:**
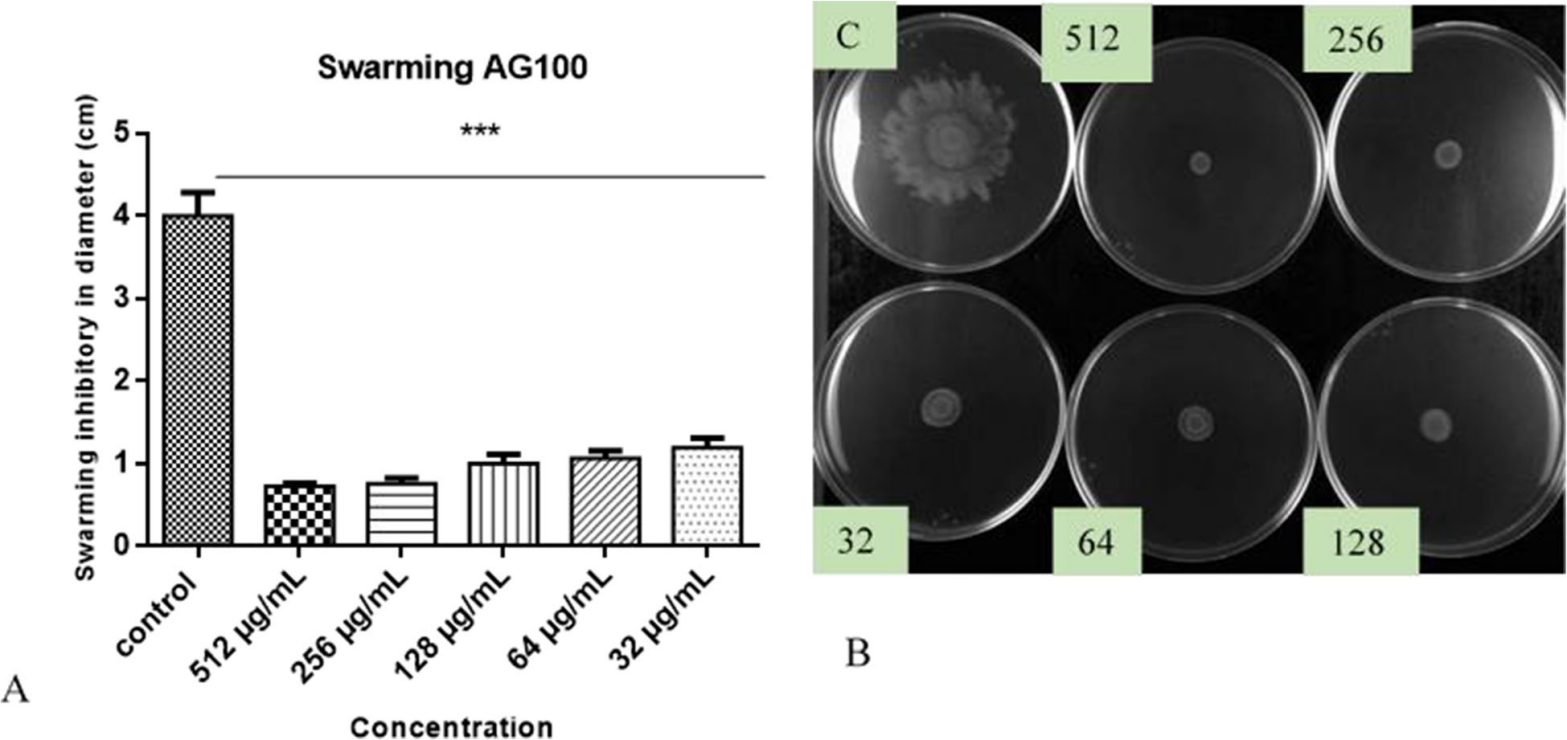
**A** Effect of HEASG on AG100 swimming motility, **B** HEASG inhibited swimming motility, respectively. Each experiment was carried using three independent cultures, and the representative values are presented. The results are expressed as mean ± SD. *n* = 3; *** indicated *p* < 0.001, ** indicated *p* < 0.01 and * indicated *p* < 0.05 versus the untreated control

The Fig. 6A shows that HEASG inhibits the swarming motility of AG100 from 32 to 512 mg/L. The results showed that with a concentration of 32 and 64 μg/mL, we obtained a swarming motility inhibition of 70 and 73% of control respectively. In contrast to swarming (Fig. 7A), HEASG at different concentrations did not affect the swimming motility comparatively the control.

## Discussion

Extract of *A. senegal* has previously shown an attractive antibacterial activity [7, 16]. In the present work the acute and subacute toxicity of the hydroethanolic extract of leaves is evaluated.

The cytotoxic activity results obtained show that the treatment of HaCaT cells with low concentrations of *Acacia senegal* leaves extracts exhibited no cytotoxic effects when the use of high concentrations (1000 μg/mL) resulted in a noticeable cytotoxicity. According to Kuete and Efferth [29], in normal cell lines the cytotoxicity of plant extracts is important or strong when the IC_50_ < 40 μg/mL; moderate cytotoxicity: 40 *μ*g/mL < IC_50_ < 120 *μ*g/mL, low cytotoxicity: 120 *μ*g/mL < IC_50_ < 400 *μ*g/mL and no cytotoxicity: IC_50_ > 400 *μ*g/mL. It is interesting to notify that the IC_50_ (1302 ± 60 μg/mL) observed by *A. senegal* HE against the normal HaCaT cell is considerably higher than 400 μg/mL making HEASG a promising candidate for future studies. Also, HEASG is able to potentialize the phenicols activities and permeabilize the bacterial outer membrane (OM) at concentrations of 32 and 64 μg/mL [16] that are non-cytotoxic in the assays reported here.

In the acute toxicity study, during the 14-day observation period, no adverse effect or mortality was observed at the single administration dose of 2000 mg/kg b.w. According to OECD guideline 423, the LD_50_ is estimated at 5000 mg/kg orally and subsequently, the plant extract corresponds to the 5th toxicity class, i.e. products with relatively low acute oral toxicity.

Likewise, the subacute treatment showed that HEASG at doses of 250, 500 and 1000 mg/kg/day during 28 days did not produce any deaths or clinical signs of toxicity. Additionally the body weight, water and food intake were not altered during the treatment period an indicator of the absence of adverse effects of drugs and chemicals under these conditions [30, 31]**.**

In the same way, the treatment with HEASG leaves did not change the biochemical parameters analyzed, except for a decrease (*P* < 0.05) in chloride serum levels in the groups treated with HEASG leaves at 250 and 500 mg/kg in both sexes. Chloride is the anion most present in serum**,** and its essential role is to maintain electroneutrality [32]. The variation in the chloride level in this study is not dose-dependent, since with the highest dose (1000 mg/kg) we have similar levels with the control group. Several studies have reported the toxicity of plant extracts on liver and kidney function and creatinine and transaminases were used as a marker of toxicity for kidney and liver, respectively [33, 34]. The preservation of the different values (creatinine, AST and ALT) indicates the absence of any adverse activity on these vital organs following the administration of the different doses of HEASG. The relative kidney and liver weights of the treated groups showed no significant differences and macroscopic examination revealed no notable damage. The other vital organs (spleen, heart, lungs, and gonads) also showed no noticeable damage and significant variation of the relative weight **(**Table 4).

Many studies have reported different types of movement such as swimming, swarming and twitching in PA01, [35, 36]. These motilities play a significant role in biofilm formation and bacterial virulence [35, 37]**.** The different types of motility are due to the presence of surface bacterial appendages*,* such as flagella and pili [38]. HEASG’s study against the motility of *P. aeruginosa* and *E. coli* showed that *A. senegal* extract inhibits both the swimming and swarming motility of PA01. However, the same extract had no effect on swimming motility but reduced the swarming motility of AG100. Several authors have shown that plant extracts or plant-derived compounds have similar impacts on bacterial motility. Jin-Hyung Lee et al. [39] demonstrated that *Carex dimorpholepis* extract and trans-resveratrol clearly reduced swimming motility and suppressed swarming motility of *E. coli* enterohemorrhagic O157: H7 (EHEC). Also, *Mallotus japonica* extract increased in swimming motility without changing swarming motility. Other authors also reported that extracts (100 μg/mL) of cranberry and pomegranate rich in proanthocyanidins (PAC) and ellagitannins respectively, blocked swarming motility, but did not block swimming motility or twitching into PA01 [40] The proanthocyanidins present in cranberries are condensed tannins composed of catechin and epicatechin monomers which are different from hydrolyzable tannins such as ellagitannins that predominate in pomegranates [41]. A recent study reports tannins, also quantifying condensed and hydrolyzable tannins in the hydroethanolic extract of the leaves of *A. senegal* [16]. The recorded anti-swarming and anti-swimming activities could be attributed to the tannins contained in the *A. senegal* extract.

On the other hand, *E. coli* is responsible for nearly 95% of urinary tract infections [42]**.** The infection is triggered by bacterial adhesion to the uroepithelium, followed by multiplication and bacterial colonization of the urinary tract [43]. A research study conducted by Amy B. Howell [44] had already attributed the anti-adhesion property to proanthocyanidins. Besides, swimming and swarming motilities positively influence biofilm development in *E. coli* and *P. aeruginosa* [45–47]**.** These results pave the way for the search for new anti-swarming and anti-swimming molecules in the treatment of certain bacterial diseases such as urinary tract infections and opportunistic diseases caused by Gram-negative bacteria.

## Conclusion

The study demonstrated that HEASG could be considered as relatively safe in terms of toxicity since no significant lethality and noticeable adverse biochemical and morphological effects are observed in acute or sub-acute toxicity studies in rats. HEASG is non-cytotoxic at concentrations able to enhance antibiotics activities and permeabilize the outer membrane of Gram-negative bacteria. The study also demonstrated the effect of HEASG on the swarming and swimming motility of PA01 and AG100. Further work is needed to precisely determine the mode of action of the active molecules. Bacterial motility is involved in biofilm and quorum formation, and also in bacterial virulence in various nosocomial infections. Further research on the efficacy of HEASG on bacterial membrane organization involved in biofilm formation and virulence will be engaged in the future.

## Supplementary Information

**Additional file 1.**

## Data Availability

The datasets generated and analyzed during the current study are available from the corresponding author on reasonable request. All data generated or analyzed during this study are included in this published article.

## Abbreviations

DMSO: Diméthylsulfoxyde
HEASG: Hydroethanolic *Acacia segenal*
LB: Luria Bertani
MDR: Multi Drug Resistant
MIC: Minimal Inhibitory Concentration
OM: Outer Membrane
CCL4: Carbon tetrachloride
AST: Aspartate aminotransferase
ALT: Alanine aminotransferase
IC_50_: 50% inhibitory concentration
LD_50_: Median lethal dose
OECD: Organization for Economic Cooperation and Development
LDL: Low density lipoprotein
HDL: High density lipoprotein
TTG: Tissue transglutaminase
TC: Total cholesterol
DEHP: Di-2- Ethylhexyl phthalate
LPO: Lipid peroxidation
OD: Optical Density
PAC: proanthocyanidins
