# Supplementary material for: Toxicity and bacterial anti-motility activities of the hydroethanolic extract of Acacia senegal (L.) Willd (Fabaceae) leaves
Source: BMC Complement Med Ther. 2021 Jun 29;21:178. doi: 10.1186/s12906-021-03348-5 (PMC8243867; doi:10.1186/s12906-021-03348-5)

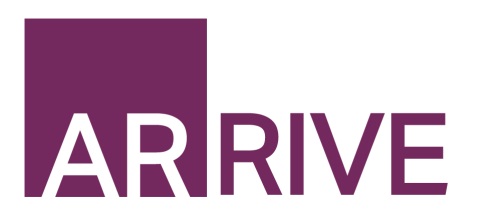


The ARRIVE Guidelines Checklist

Animal Research: Reporting In Vivo Experiments

Carol Kilkenny^1^, William J Browne^2^, Innes C Cuthill^3^, Michael Emerson^4^ and Douglas G Altman^5^

*^1^The National Centre for the Replacement, Refinement and Reduction of Animals in Research, London, UK, ^2^School of Veterinary Science, University of Bristol, Bristol, UK, ^3^School of Biological Sciences, University of Bristol, Bristol, UK, ^4^National Heart and Lung Institute, Imperial College London, UK, ^5^Centre for Statistics in Medicine, University of Oxford, Oxford, UK.*

|  | ITEM | RECOMMENDATION | Section/ Paragraph | |  |
| --- | --- | --- | --- | --- | --- |
| 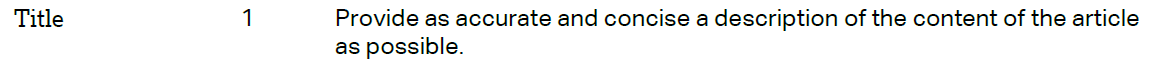 | | | | 1. Title | |
| 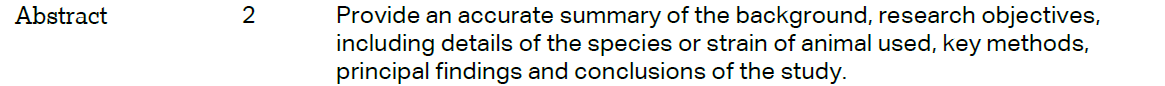 | | | | 2. Abstract | |
| INTRODUCTION | | | |  | |
| 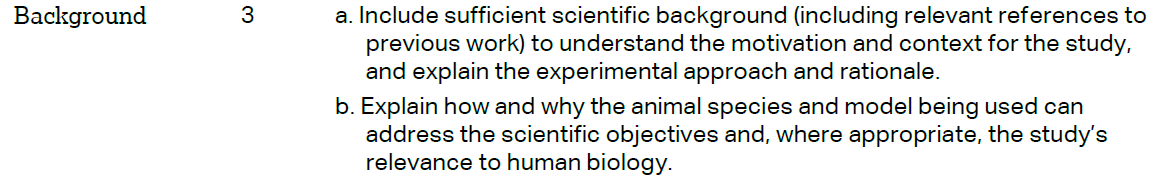 | | | | 3a. Background, Paragraph 2  3b.Background, Paragraph 3 | |
| 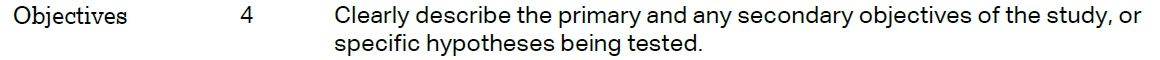 | | | | 4. Objective, Last paragraph | |
| METHODS | | | |  | |
| 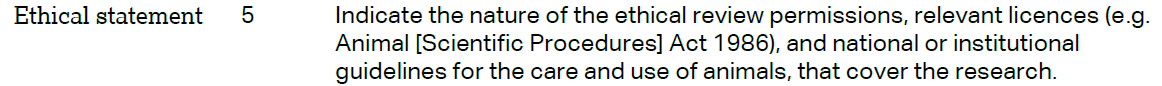 | | | | 5. Methods section, Experimental Animals | |
| 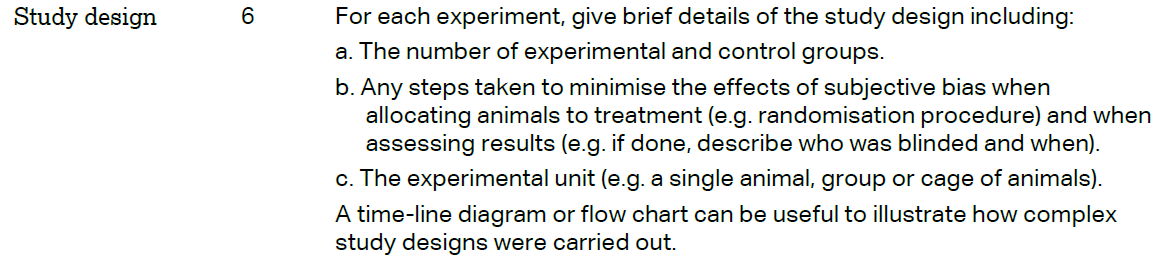 | | | | 6a. Materials and Methods section, animals subsection  6b. Methods section   - Acute toxicity test subsection - Sub-acute toxicity study subsection - Collection of blood samples subsection Sub-acute toxicity test - Assessment of biochemical parameters subsection - Determination of the Relative Weight of Organs   6c. Methods section   - Acute toxicity test subsection (6 females mice for the test group and 3 females mice for the control group) - Sub-acute toxicity study subsection (4 groups of 10 animals per group: 5 females and 5 males). Brief, 6 group test and 2 group control | |
| 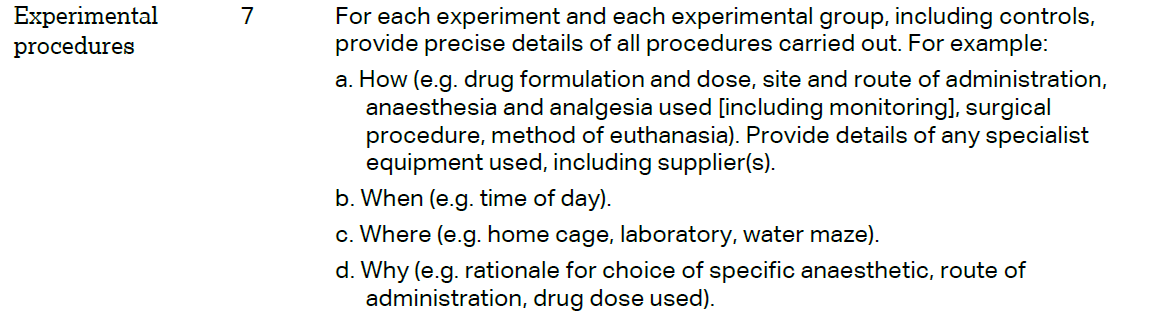 | | | | 7a. Methods section   - Acute toxicity test subsection - Sub-acute toxicity study subsection   7b. Methods section   - Acute toxicity test subsection - Sub-acute toxicity study subsection   7c. Methods section, Animals subsection  7d. Methods section   - Acute toxicity test subsection - Sub-acute toxicity study subsection | |
| 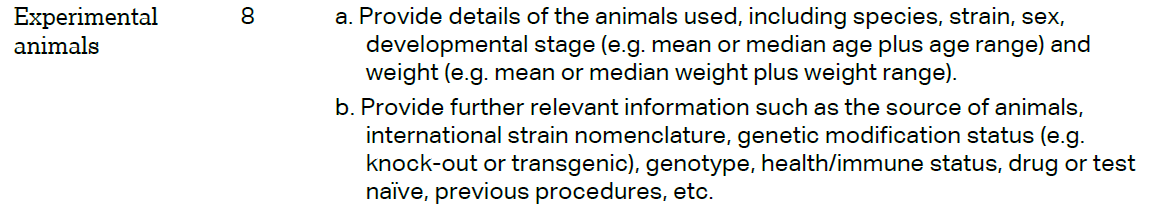 | | | | 8a. Methods section, experimental animals subsection   - Acute toxicity test subsection - Sub-acute toxicity study subsection   8b. Methods section, experimental animals subsection  Acute oral toxicity test subsection (animals) | |

The ARRIVE guidelines. Originally published in *PLoS Biology*, June 2010^1^

| 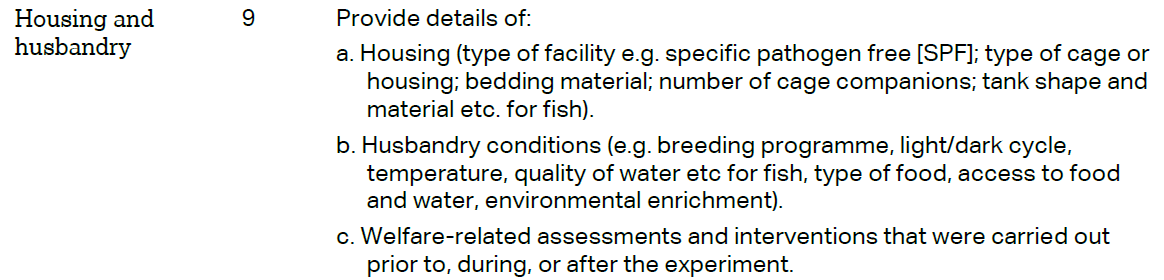 | 9a. Methods section, experimental animals subsection   - Acute toxicity test subsection - Sub-acute toxicity study subsection   9b. Methods section, experimental animals subsection  9c. Methods section, experimental animals subsection   - Acute toxicity test subsection - Sub-acute toxicity study subsection | |
| --- | --- | --- |
| 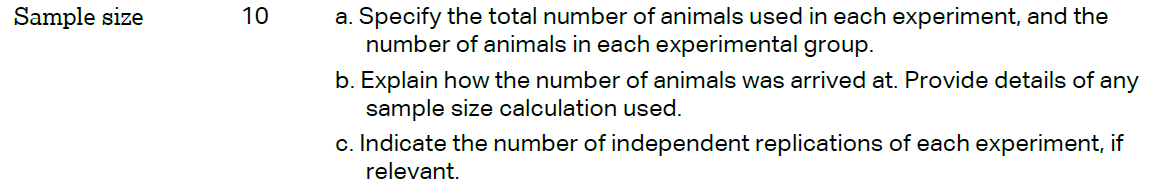 | 10a. Method section, experimental animals subsection  10b. Methods section:   - Acute toxicity test subsection - Sub-acute toxicity study subsection   10c. Methods section, experimental animals subsection   - Acute toxicity test subsection - Sub-acute toxicity study subsection | |
| 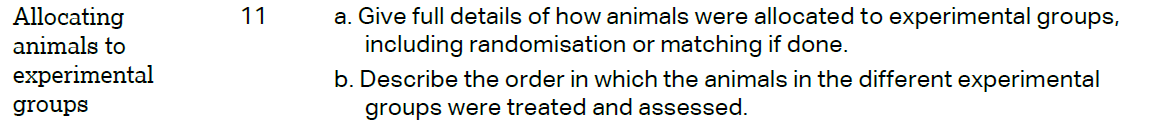 | 11a. Methods section, experimental animals subsection   - Acute toxicity test subsection - Sub-acute toxicity study subsection   11b. Methods section, experimental animals subsection   - Acute toxicity test subsection - Sub-acute toxicity study subsection | |
| 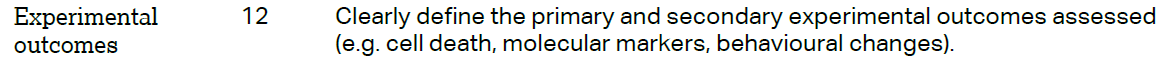 | 12. Methods section, experimental animals subsection   - Acute toxicity test subsection - Sub-acute toxicity study subsection - Collection of blood samples subsection - Assessment of biochemical parameters subsection | |
| 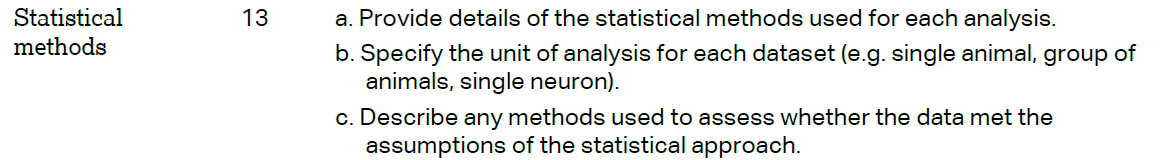 | 13a. Methods section: statistical analysis subsection  13b. Methods section: statistical analysis subsection  13c. Methods section: statistical analysis subsection | |
| RESULTS |  | |
| 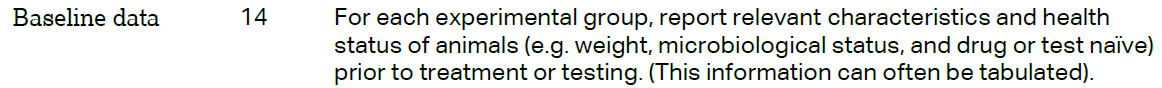 | 14. Results section, Tables 2-5 (dose in mg/kg) and figure 5 & 6 | |
| 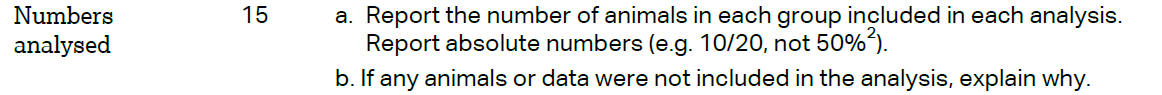 | 15a.Results section   - Acute toxicity test subsection - Sub-acute toxicity study subsection - Effect of HEASG on organs weights in rats subsection - Effect of HEASG on biochemical parameters of rats subsection   15b. Not applicable | |
| 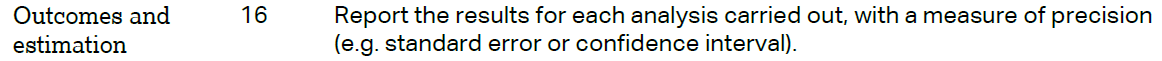 | 16. Results section, Tables 2-5 and Figures 1-7 | |
| 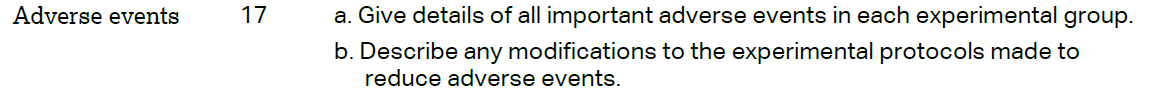 | 17. Not applicable | |
| DISCUSSION |  | |
| 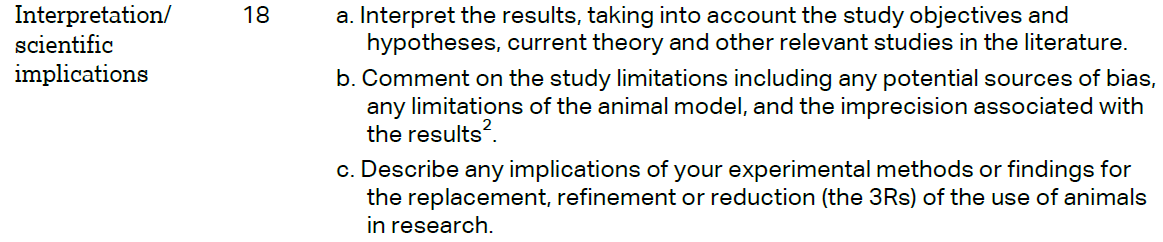 | 18. Discussion section   - Acute toxicity test subsection - Sub-acute toxicity study subsection | |
| 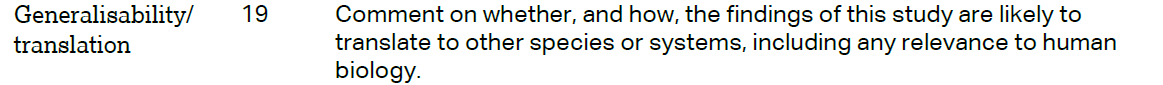 | 19. Not applicable | |
| 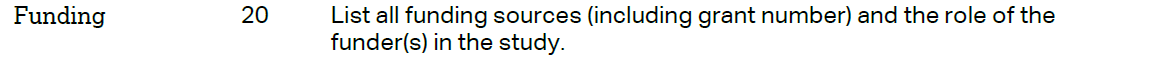 | | 20. Funding section |


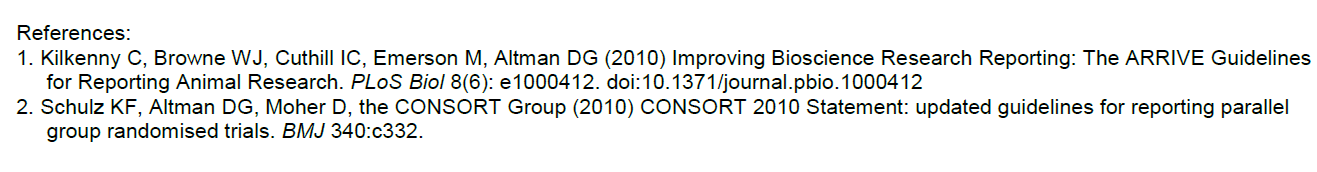

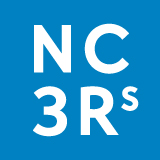

Supplement: Supplementary file 1 — Additional file 1. [file 12906_2021_3348_MOESM1_ESM.docx]
